# Sequence Variants of the *Phytophthora sojae* RXLR Effector Avr3a/5 Are Differentially Recognized by *Rps*3a and *Rps*5 in Soybean

**DOI:** 10.1371/journal.pone.0020172

**Published:** 2011-07-14

**Authors:** Suomeng Dong, Dan Yu, Linkai Cui, Dinah Qutob, Jennifer Tedman-Jones, Shiv D. Kale, Brett M. Tyler, Yuanchao Wang, Mark Gijzen

**Affiliations:** 1 Agriculture and Agri-Food Canada, London, Ontario, Canada; 2 Department of Plant Pathology, Nanjing Agricultural University, Nanjing, China; 3 Virginia Bioinformatics Institute, Virginia Tech, Blacksburg, Virginia, United States of America; University of Wisconsin-Milwaukee, United States of America

## Abstract

The perception of *Phytophthora sojae* avirulence (*Avr*) gene products by corresponding soybean resistance (*Rps*) gene products causes effector triggered immunity. Past studies have shown that the *Avr3a* and *Avr5* genes of *P. sojae* are genetically linked, and the *Avr3a* gene encoding a secreted RXLR effector protein was recently identified. We now provide evidence that *Avr3a* and *Avr5* are allelic. Genetic mapping data from F_2_ progeny indicates that *Avr3a* and *Avr5* co-segregate, and haplotype analysis of *P. sojae* strain collections reveal sequence and transcriptional polymorphisms that are consistent with a single genetic locus encoding *Avr3a/5*. Transformation of *P. sojae* and transient expression in soybean were performed to test how *Avr3a/5* alleles interact with soybean *Rps*3a and *Rps*5. Over-expression of *Avr3a/5* in a *P. sojae* strain that is normally virulent on *Rps*3a and *Rps*5 results in avirulence to *Rps*3a and *Rps*5; whereas silencing of *Avr3a/5* causes gain of virulence in a *P. sojae* strain that is normally avirulent on *Rps*3a and *Rps*5 soybean lines. Transient expression and co-bombardment with a reporter gene confirms that *Avr3a/5* triggers cell death in *Rps*5 soybean leaves in an appropriate allele-specific manner. Sequence analysis of the *Avr3a/5* gene identifies crucial residues in the effector domain that distinguish recognition by *Rps*3a and *Rps*5.

## Introduction

*Phytophthora sojae* is an oomycete and a plant pathogen that infects soybean. It is one of more than 80 species of *Phytophthora* that cause destructive diseases of a large range of agriculturally and ornamentally important plants and native species in forests and natural ecosystems. *P.sojae* is a soil-borne pathogen that is highly host-specific to soybean, causing damping-off of seedlings and root rot of older plants, with an estimated annual cost of $1–2 billion worldwide [1], [2]. Management of the disease relies in part on the development and proper deployment of soybean varieties with cultivar-specific resistance traits.

Historically, the gene-for-gene model provided a framework to interpret the interaction between cultivar-specific resistance in a host plant and strain-specific virulence in a pathogen [3]. This concept established the association between genes conditioning avirulence in the pathogen and corresponding genes conferring resistance in the host. Now, we understand that pathogen avirulence (*Avr*) genes encode effectors that normally enable pathogen growth on host plants in the absence of appropriate host resistance (*R*) genes [4]. Host plant *R* genes constitute elements of the plant immune system that cause effector-triggered immunity in the presence of corresponding pathogen Avr factors.

The identification of oomycete *Avr* genes has a recent history that follows the development of advanced molecular and genetic technologies. Inheritance studies of *Avr* genes in *P. sojae* provided a basis for genetic mapping in F_2_ populations [5], [6]. This work established that at least three pairs of *P. sojae Avr* genes are genetically linked, specifically *Avr1b* and *Avr1k*, *Avr3a* and *Avr5*, and *Avr4* and *Avr6* [7], [8], [9], [10]. The *Avr4* and *Avr6* genes have since been identified and found to be encoded by a single locus, *Avr4/6* [11]. Other oomycete *Avr* genes that have been identified include *Avr1a*, *Avr1b*, *Avr3a*, and *Avr3c* from *P. sojae* [12], [13], [14], *Avr3a*, *Avr4*, *Avr-blb1* and *Avr-blb2* from *Phytophthora infestans* [15], [16], [17], [18], and *ATR1*, *ATR5*, and *ATR13* from *Hyaloperonospora arabidopsidis* [19], [20], [21]. With the exception of *ATR5*, each of these oomycete *Avr* genes encode predicted secreted proteins with a conserved motif consisting of RXLR (Arg-any amino acid-Leu-Arg) and variable EER (Glu-Glu-Arg) sequences that occur downstream from the amino terminal signal peptide. The *ATR5* sequence is predicted to encode a secreted protein that includes an EER and an RGD (Arg-Gly-Asp) motif, but no canonical RXLR motif. It is believed that the RXLR, EER, and RGD motifs are responsible for targeting the effectors into host cells [20], [22], [23], [24]. The RXLR effectors comprise large and rapidly evolving gene families in *Phytophthora* species. The best-studied species include *P. infestans* and *P. sojae*, with an estimated 563 and 396 RXLR effectors, respectively [25], [26], [27].

The identification of 12 different oomycete *Avr* genes and the finding that all encode secreted effectors targeted for delivery into host cells has opened up new avenues of investigation. The momentum of research has shifted towards functional studies of the effectors themselves and their mechanism of host-targeting. Nonetheless, the identification and characterization of new Avr factors remains relevant, since they are the crucial virulence determinants in cultivar-specific interactions. Perhaps new patterns will emerge among RXLR effectors that operate as Avr factors. On the practical side, the identification of *P. sojae Avr* genes will provide important tools for breeding and disease management in soybean, which is among the economically most important crops on the globe.

In this paper, we show that strain-specific virulence of *P. sojae* to soybean plants carrying the *Rps*3a or *Rps*5 resistance genes is determined by a single genetic locus, *Avr3a/5*. Based upon genetic mapping data, genome sequence assemblies, haplotype analysis, and the predicted RXLR secretome of *P.sojae*, we hypothesized that the *Avr3a* and *Avr5* genes are alleles. This was confirmed by functional tests based upon transient expression in soybean and stable transformation of *P. sojae*. Variation in amino acid sequence within the effector domain of Avr3a/5 determines recognition by *Rps*3a or by *Rps*5.

## Results

### Genetic mapping and haplotype analysis suggest that *Avr3a* and *Avr5* are alleles of a single locus

**Figure 1:**
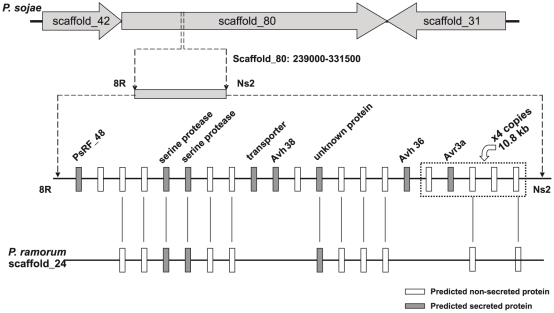
Physical map of the *P. sojae Avr3a* and *Avr5* region. Shown are the positions of predicted genes including candidate RXLR effector genes, and synteny with *P. ramorum*. In *P. sojae* strain P6497, *Avr3a* is embedded in a 10.8 kb DNA segment that is present in a tandem array of four copies, which is shown as a box in this illustration (not drawn to scale). Scaffold designations are from v1.0 of the genome sequence.

Previous studies have shown that *Avr3a* and *Avr5* are genetically linked [6], . The most recent work, showing that *Avr3a* and *Avr5* precisely co-segregate in 100 F_2_ progeny, resulted in the identification of *Avr3a* [14]. Based upon fine genetic mapping of *Avr5*, this gene is predicted to occur within a 78 kb region in scaffold_80 delineated by the molecular markers named ‘Ns2’ and ‘8R’, as shown in Figure 1. To identify candidates for *Avr5*, predicted RXLR effectors (named *Avh* genes) and other secreted proteins within this region were catalogued. By examining this space and comparing it with a syntenic region from the *Phytophthora ramorum* genome assembly, we determined that three different genes encoding predicted RXLR effectors provided the most plausible candidates for *Avr5*. Thus, *Avh*38, *Avh*36, and *Avr3a* are possible candidate effector genes for *Avr5*.

Sequence and transcript analyses of these candidate genes were conducted on *P. sojae* strains P6497 and P7064 in order to detect any polymorphisms because these two strains are different in virulence characteristics. *P. sojae* P6497 is avirulent on *Rps*3a and *Rps*5 while P7064 is virulent on *Rps*3a and *Rps*5. The results are summarized in Table 1. This shows that *Avh*38 and *Avh*36 sequences are identical in *P. sojae* strains P6497 and P7064, and that there are no transcriptional polymorphisms for these genes between the two strains. Expression of *Avh*38 occurs in virulent and avirulent strains, whereas *Avh*36 transcripts could not be detected in either strain. In contrast, the *Avr3a* sequence displayed copy number polymorphisms, amino acid changes, and expression differences between *P. sojae* strains P6497 and P7064, as we previously reported [14]. Thus, *Avr3a* remains the best candidate for *Avr5*.

**Table 1:** Summary of *Avr5* candidate genes in *P.sojae* strains P6497 and P7064^1^.

Gene	Sequence polymorphisms	Transcript polymorphisms
*Avh38*	None apparent	No difference; expression detected in both strains
*Avh36*	None apparent	No difference; no expression detected in both strains
*Avr3a*	DNA sequence differences causing promoter changes and amino acid substitutions, and copy number variation	Polymorphic; expressed in P6497 but not in P7064

1 *P. sojae* strain P6497 is avirulent while strain P7064 is virulent, on soybean plants with the *Rps*5 resistance gene.

**Figure 2:**
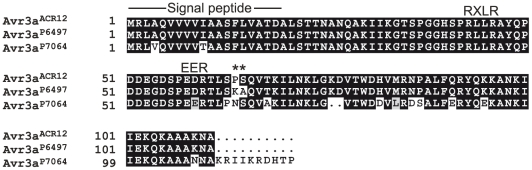
Predicted amino acid sequences of *Avr3a* alleles from three strains of *P. sojae*. The position of the predicted signal peptide and the RXLR and EER host-targeting sequences are shown. The deduced Avr3a protein sequence in *P. sojae* strains P6497 and ACR12 differs by two amino acids, as indicted by the asterisks. The *Avr3a* genes from strains P6497 and ACR12 are transcribed whereas no transcripts can be detected for *Avr3a* from P7064.

The *Avr3a* locus is highly polymorphic among *P. sojae* strains, as summarized in Table 2. Transcriptional differences among strains are sufficient to account for virulence differences on *Rps*3a but not on *Rps*5. This is because at least three strains (ACR12, P7076, and ACR20) express *Avr3a* but are virulent on *Rps*5. These three strains show differential virulence towards *Rps*3a and *Rps*5. Thus, gain of virulence on *Rps*5 in these strains could result from sequence differences, since Avr3a^ACR12^ differs by two amino acids from Avr3a^P6497^. Compared to the reference strain (P6497) the sequence of Avr3a^ACR12^ shows changes to adjacent amino acids K64P and A65S within the effector domain of the predicted protein, as shown in Figure 2. The predicted amino acid sequence of Avr3a^P7064^ is also shown for comparison, but *Avr3a^P7064^* is likely a pseudogene because no transcripts can be detected for this gene in strains that carry it.

**Table 2:** Virulence phenotypes and haplotype analysis of *P. sojae* strains.

*P. sojae* strain^1^	Virulence^2^ *Rps*3a	Virulence^2^ *Rps*5	*Avr3a* copy number^3^	*Avr3a* mRNA^4^	PromoterINDEL^5^	*Avr3a* sequence^6^
48FPA18	A	A	4	+	−	P6497
P6497	A	A	4	+	−	P6497
25MEX4	A	A	4	+	−	P6497
ACR8	A	A	4	+	−	P6497
ACR9	A	A	4	+	−	P6497
ACR11	A	A	4	+	−	P6497
ACR25	A	A	4	+	−	P6497
ACR10	V	V	4	−	−	P6497
ACR16	V	V	4	−	−	P6497
ACR12	A	V	1	+	−	ACR12
P7076	A	V	1	+	−	ACR12
ACR20	A	V	1	+	−	ACR12
P7064	V	V	1	−	+	P7064
ACR17	V	V	1	−	+	P7064
P7074	V	V	1	−	+	P7064
ACR21	V	V	1	−	+	P7064
ACR24	V	V	1	−	+	P7064

1 Origins of P. sojae strains are provided in .

2 A, Avirulent; V, virulent.

3 The *Avr3a* gene occurs in a 10.8 kb DNA segment that is present either as one copy or as four tandemly-arrayed copies, as indicated.

4 Positive (+) or negative (−) for expression of *Avr3a* transcripts, as determined by RT-PCR.

5 Insertions and deletions (INDEL) within the promoter region of *Avr3a* gene, as illustrated in Figure S1.

6 Three different DNA sequences for the *Avr3a* open reading frame, indicated according to the representative strain. GenBank accession numbers are as follows: EF587759 (P6497), JF412456 (ACR12), and JF433921 (P7064).

### Transient expression of *Avr3a^P6497^* triggers cell death in *Rps*5 soybean leaves

**Figure 3:**
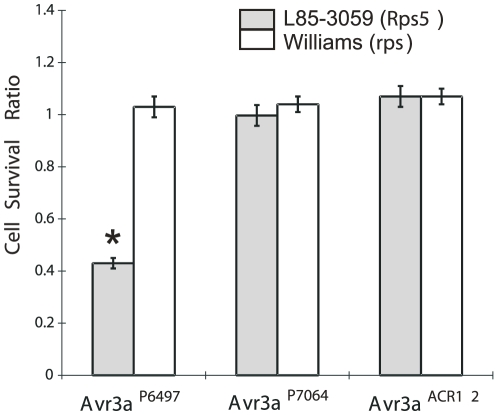
Expression of *Avr3a^P6497^* in *Rps*5 soybean plants can trigger cell death. Results from a co-bombardment assay are shown. The ratio of GUS-positive blue spots following co-bombardment with *Avr3a^P6497^*, *Avr3a^P7064^* and *Avr3a^ACR12^* compared with the empty vector DNA. Expression constructs for *Avr3a* alleles were without signal peptides. Soybean lines Williams (*rps*) and L85–3059 (*Rps*5) are genetic isolines. Bars represent standard errors from 12–14 replicates each. An asterisk (*) indicates a significant difference (p<0.001) by the Wilcoxon rank sum test.

The proteins encoded by *P. sojae Avr* genes trigger defense responses and cell death when they are recognized by soybean *Rps* gene products. In order to test whether the various alleles of *Avr3a* can interact functionally with *Rps*5, a co-bombardment and transient expression test was performed. Plasmid constructs directing expression of *Avr3a^P6497^*, *Avr3a^P7064^*, and *Avr3a^ACR12^* without native signal peptide were introduced into leaves of an *Rps*5 soybean cultivar together with a reporter gene (beta-glucuronidase, GUS) to measure cell viability, as shown in Figure 3 (representative photographs shown in Figure S2). Results indicate that *Avr3a^P6497^* interacts with *Rps*5, as evidenced by a two-fold reduction in GUS staining in *Rps*5 plants compared to control plants without this resistance gene. In contrast, co-transformation with either *Avr3a^P7064^* or *Avr3a^ACR12^* does not reduce GUS expression by this assay, regardless of the presence of *Rps*5. Past results have shown that *Rps*3a interacts with *Avr3a^P6497^* and *Avr3a^ACR12^* [14]. Thus, the co-bombardment assays provide functional evidence for the hypothesis that *Avr3a* and *Avr5* are alleles.

### Over-expression of *Avr3a^P6497^* in *P. sojae* strain P7074 causes gain of avirulence in the presence of *Rps*3a and *Rps*5

**Figure 4:**
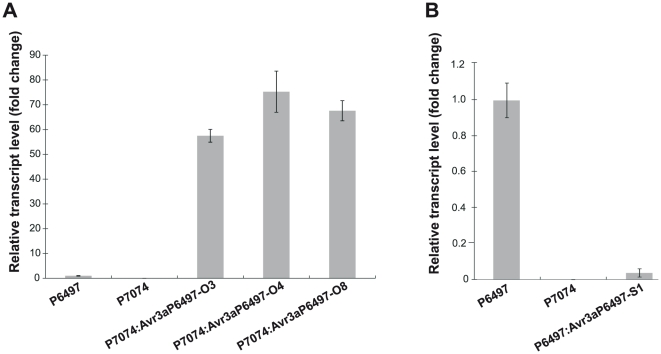
Expression levels of *Avr3a* in wild-type and transgenic strains of *P. sojae*. (A) Relative expression of *Avr3a* in mycelia cultures of wild-type strains P6497 and P7074, and transgenic over-expressing strains P7074:Avr3a^P6497^-O3, P7074:Avr3a^P6497^-O4, and P7074:Avr3a^P6497^-O8. Expression was measured by quantitative real time PCR and normalized to the level in wild-type P6497. (B) Relative expression of *Avr3a* in mycelia cultures of wild-type strains P6497 and P7074, and transgenic silenced strain P6497:Avr3a^P6497^-S1. Expression was measured by quantitative real time PCR and normalized to the level in wild-type P6497. Bars indicate standard errors from three independent replicates.

In order to obtain additional evidence that the *Avr3a^P6497^* gene product interacts with *Rps*5, we transferred *Avr3a^P6497^* into protoplasts of *P.sojae* strain P7074 to produce stable transformants. The strain P7074 carries an *Avr3a* allele identical in sequence to *Avr3a^P7064^* and is normally virulent on *Rps*3a and *Rps*5. Three stable transformants (P7074:Avr3a^P6497^-O3, -O4, and -O8) with high expression levels of the transgene *Avr3a^P6497^* were recovered, as shown in Figure 4A. These results show that ectopic expression of *Avr3a^P6497^* with the heterologous *Ham34* promoter from *Bremia lactucae* causes mRNA levels to be several-fold greater than in wild type strains that express this gene off the native promoter.

**Figure 5:**
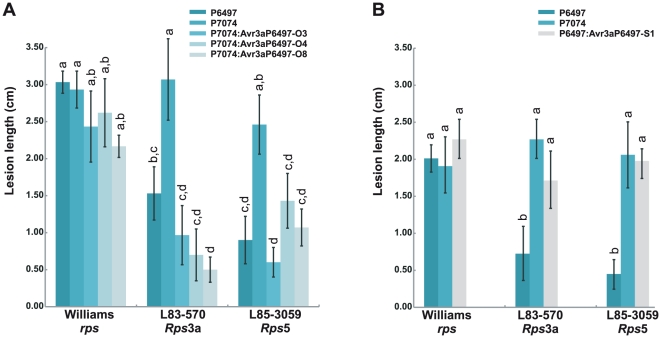
Disease lesion lengths in etiolated soybean hypocotyls infected with zoospores of wild-type and *Avr3a* transformed strains of *P. sojae*, 36 h after inoculation. Mean and standard error from at least 20 measurements are shown in each case, with results from Duncan's multiple range test (1% significance) indicated above each column. (A) Ectopic over-expression of *Avr3a^P6497^* causes loss of virulence to *Rps*3a and *Rps*5 in strain P7074. Shown are results from wild-type strains P6497 and P7074, and transgenic over-expressing strains P7074:Avr3a^P6497^-O3, P7074:Avr3a^P6497^-O4, and P7074:Avr3a^P6497^-O8. (B) Silencing of *Avr3a^P6497^* causes gain of specific virulence in the presence of *Rps*3a and *Rps*5 in strain P6497. Shown are results from wild-type strains P6497 and P7074, and transgenic silenced strain P6497:Avr3a^P6497^-S1.

Virulence assays of the wild-type and transformed *P. sojae* strains were performed by two different methods, consisting of stem inoculations of mycelia on light-grown seedlings and zoospore inoculations of etiolated hypocotyls. Results from stem inoculations of light-grown seedlings are shown in Table 3 (representative photographs shown in Figure S2). As expected, wild-type *P. sojae* strain P7074 is virulent in the presence of *Rps*3a and *Rps*5 whereas P6497 is avirulent on each of these genes. Inoculation with *Avr3a^P6497^*-transformed strains produced mostly resistant reactions on *Rps*5 and *Rps*3a soybean plants, indicating a gain of specific avirulence due to transgenes. The zoospore inoculation test yielded results similar to the stem inoculation assay with mycelia, as shown in Figure 5A. In the zoospore assay, lesion lengths provide a measure of virulence. Inoculation of *Rps*5 and *Rps*3a plants with zoospores from *Avr3a^P6497^*-transformed strains (P7074:Avr3a^P6497^-O3, -O4 and -O8), or from wild-type strain P6497, resulted in dark-brown necrosis and relatively small lesion sizes, as expected for avirulent or resistant interactions. In contrast, the wild-type strain P7074 caused spreading water-soaked lesions that are characteristic of virulent or susceptible interactions. Thus, we conclude that heterologous expression of *Avr3a^P6497^* in *P. sojae* P7074 causes gain of avirulence in the presence of *Rps*3a and *Rps*5.

**Table 3:** Virulence characteristics of *P. sojae* wild-type strains P6497 and P7074 and *Avr3a*-transformed strains.

*P. sojae* strain^1^	Disease outcome on soybean isolines^2^		
	Williams (*rps*)	L83–570 (*Rps*3a)	L85–3059 (*Rps*5)
P6497	V (4/42)	A (69/69)*	A (54/54)*
P7074	V (0/31)	V (4/66)	V (3/63)
P7074:Avr3a^P6497^-O3	V (4/31)	A (27/29)*	A (23/28)*
P7074:Avr3a^P6497^-O4	V (2/29)	A (21/27)*	A (21/41)*
P7074:Avr3a^P6497^-O8	V (0/18)	A (25/25)*	A (20/29)*
P6497:Avr3a^P6497^-S1	V (3/58)	V (0/46)	I (14/61)*

1 Two wild-type strains (P6497 and P7074) and four transformed strains, either over-expressing *Avr3a^P6497^* (-O3, -O4, and -O8) or silenced for expression of *Avr3a^P6497^* (-S1) are shown.

2 A, avirulent; I, intermediate; V, virulent. Results from stem inoculation of light-grown plants, number of surviving plants/total plants shown in parenthesis. Fischer's exact test was performed to compare the outcome on Williams (*rps*) to that on the *Rps*3a and *Rps*5 isolines, for each strain; asterisks indicate significant difference (p<0.05).

### Silencing of *Avr3a^P6497^* in *P. sojae* strain P6497 causes loss of avirulence in the presence of *Rps*3a and *Rps*5

To further test the interaction of *Avr3a^P6497^* and *Rps*5, we obtained a gene-silenced transformed strain of *P. sojae* P6497. Levels of the *Avr3a^P6497^* transcript are severely reduced in the gene-silenced strain compared to the wild-type strain P6497, as shown in Figure 4B (representative photographs shown in Figure S2). Stem inoculation of light-grown seedlings was performed to test the virulence of the gene-silenced strain compared to wild-type controls, as shown in Table 3. These results show that soybean plants containing *Rps*3a and *Rps*5 were mostly killed by strain P6497:Avr3a^P6497^-S1, whereas plants inoculated with the wild type strain P6497 survived. Furthermore, as shown in Figure 5B, the *Avr3a^P6497^*-silenced strain (P6497:Avr3a^P6497^-S1) produced large water-soaked lesions by the zoospore inoculation assay, similar to strain P7074 which is known to be virulent in the presence of *Rps*3a and *Rps*5. Thus, the silencing of *Avr3a^P6497^* expression in P6497 results in gain of virulence in the presence of *Rps*3a and *Rps*5.

## Discussion

To understand effector-triggered immunity in plants, it is useful to consider the hypothesis that pathogen Avr factors simply represent effectors that have had *R* genes targeted to them and selected for in host plant populations. The completion of the *P. sojae* genome sequence and the discovery of RXLR effectors have facilitated *Avr* gene identification for this and other oomycete plant pathogens. Nonetheless, *Avr* gene identification still requires substantial work to create short-lists of candidate genes and to test their interaction with soybean *Rps* genes. In this paper, we identify the *Avr5* gene from *P. sojae*. We provide evidence that *Avr3a* and *Avr5* are alleles and represent a single genetic locus, *Avr3a/5*.

Previous studies have demonstrated that the RXLR motif is necessary and sufficient for delivery of proteins into plant cells [22], [23]. Results from our co-bombardment assays indicate that *Avr3a/5* is able to trigger cell death in *Rps*5 plants in an appropriate allele-specific manner, when expressed without a signal peptide. Thus, we conclude that recognition of *Avr5* occurs within the plant cell and that *Rps*5 is a cytoplasmic *R*-gene.

We have recently described the organization of the *Avr3a/5* gene and the haplotypes of the locus among *P. sojae* strains [14], but it is worthwhile to briefly review these features here. The *Avr3a/5* gene is embedded in a 10.8 kb DNA segment that includes four other predicted genes. *P. sojae* strains may possess one or four copies of this 10.8 kb segment arranged in a tandem array. Gain of virulence on *Rps*3a is caused by transcriptional differences between strains. However, the underlying causes of the transcriptional polymorphisms appear to differ among the strains. No transcripts of *Avr3a/5* can be detected in *P. sojae* strains ACR10 and ACR16 but nevertheless these strains possess a tandem array of four *Avr3a/5* copies that is identical in nucleotide sequence to strains that are transcriptionally active, such as P6497. In other *P. sojae* strains that do not appear to express the *Avr3a/5* gene, such as P7064, *Avr3a/5* is present as a single copy gene with rearrangements (insertions and deletions) in the promoter region, compared to the reference strain P6497 (Figure S1). Our present hypothesis is that the lack of *Avr3a/5* transcripts in strains like ACR10 is an epigenetic effect caused by small RNA mediated gene silencing, whereas in strains like P7064 it is caused by a failure of transcription due to the changes present in the promoter. These theories will require further experimentation to test their validity.

Regardless of the mechanism, it is clear that the loss of *Avr3a/5* transcripts in *P. sojae* causes gain of specific virulence in the presence of either *Rps*3a or *Rps*5. Although silencing of *Avr3a/5* in transformed *P. sojae* strain P6497:Avr3a^P6497^-S1 does not gain full virulence on light-grown seedlings containing *Rps*5, this might result from some residual transcription and expression of Avr3a/5 compared to P7074; presumably this level is not high enough to trigger a response in the presence of *Rps*3a. Silencing of *Avr3a/5* did not cause loss of general virulence on Williams, suggesting that *Avr3a/5* is dispensable for general virulence. Over-expression of *Avr3a/5* in *P. sojae* strains that do not normally express this gene caused the expected loss of specific virulence in the presence of *Rps*3a or *Rps*5. However, the lines over-expressing *Avr3a/5* retained partial virulence against light-grown seedlings containing *Rps*5, but not those containing *Rps*3a. Possibly the resistance conferred by *Rps*5 was partially compromised by the unusually high level of *Avr3a/5* transcripts in the transformed strains or by other effectors in the genetic background of P7074.

We have also shown differential recognition of *Avr3a/5* alleles by *Rps*3a and *Rps*5. Crucial residues of the Avr3a/5 protein that are required for recognition by *Rps*5 were identified. Thus, the K64P and A65S changes result in evasion of recognition by *Rps*5 but not *Rps*3a. Isolates of *P. sojae* with reciprocal characteristics, displaying specific virulence in the presence of *Rps*3a but not *Rps*5, have been reported but were not available for the present study [28], [29], [30]. Field surveys of *P. sojae* pathotypes demonstrate that strains showing virulence on *Rps*3a but not *Rps*5 may exist but nevertheless are rare, as summarized in Table 4. Thus, it is possible that there are additional alleles of the *Avr3a/5* locus that are differentially recognized by *Rps*5 but not *Rps*3a. In order to resolve this, it will be necessary to isolate the strains, confirm their virulence phenotypes, determine the *Avr3a/5* gene sequences, and test their functional interaction with *Rps*3a and *Rps*5.

**Table 4:** Virulence phenotypes in the presence of *Rps*3a and *Rps*5 of *P. sojae* field isolates, from three different studies.

Location	Total isolates	Virulence phenotype^1^				Reference
		A-*Rps*3a A-*Rps*5	V-*Rps*3a A-*Rps*5	A-*Rps*3a V-*Rps*5	V-*Rps*3a V-*Rps*5	
	*n*	%	%	%	%	
Michigan	67	27	6	36	31	Kaitany et al., 2001
Ohio	289	58	4	16	21	Dorrance et al., 2003
China	75	15	3	56	27	Cui et al., 2010

1 A, Avirulent; V, virulent.

To conclude, we have shown that *Avr3a* and *Avr5* from *P. sojae* represent a single gene, *Avr3a/5*. This highly polymorphic locus displays copy number variation, sequence polymorphisms, and transcriptional differences among *P. sojae* strains. The identification of the *P. sojae Avr3a/5* gene will assist soybean breeding programs and pathogen diagnostics, and will lead to more rational disease control measures. This is important because root rot caused by *P. sojae* is among the most destructive diseases that challenges soybean producers.

## Materials and Methods

### *Phytophthora sojae* isolates, manipulation, plant materials and virulence tests

Strains of *Phytophthora sojae* were from the collection at Agriculture and Agri-Food Canada, London, ON. Original sources are provided in Table S1. *P. sojae* isolates were routinely grown on vegetable juice (V8) agar. Transformation of *P. sojae* was based on published protocols [31], [32], as described below. Genomic PCR screening of all putative transformed strains was performed with CE+p35 F/R primers. A complete primer list is provided in Table S2.

Soybean (*Glycine max*) cultivars Williams (*rps*) and the Williams isoline L83–570 (*Rps*3a) and L85–3059 (*Rps*5) used to evaluate the virulence of *P. sojae* cultures were from the collections at Agriculture and Agri-Food Canada (London, Ontario) or Virginia Tech. Stem inoculation of light grown plants was as described [13]. Inoculation of etiolated hypocotyls with *P. sojae* zoospores was carried out as described [33], [34]. Each treatment contained at least 20 plants. All inoculated seedlings were incubated in the dark at 25°C under 80% humidity. The virulence tests were repeated at least three times, independently. Fischer's exact test or Duncan's multiple range test were performed to determine significance of the results.

### Generation of F_1_ and F_2_ progeny

Cultures of F_1_ hybrids were derived from crosses of P6497×P7064. Oospores from were produced by co-cultivation of the parental strains on 2.5% (v/v) vegetable juice (V8) agar plates supplemented with 10 mg/ml beta-sitosterol. Cultures were kept at 25°C in darkness for at least 20 d to produce mature oospores. Oospores were isolated by placing a mature culture in a sterile blender (Waring) with 100 ml of 4°C sterile distilled water (SDW). The culture was macerated for 2 min and sieved twice through a sterile 75 mm nylon mesh. The filtrate was collected in 50 ml conical tubes and frozen at −20°C to kill hyphae. After 24 hr, the samples were partially thawed at 45°C for 10 min and re-filtered through a sterile 75 mm nylon mesh. The filtrate was centrifuged at 2500×*g* for 10 min to pellet the oospores. Beta-glucuronidase was added to the oospore and water suspension to a final concentration of 2000 U/ml. The mixture was incubated at 37°C for 16 h. Oospores were washed twice with SDW prior to re-suspending the pellet in 2 to 5 ml of SDW. Approximately 500 oospores were spread onto 9 cm 1.5% (w/v) water agar plates supplemented with beta-sitosterol (10 mg/ml) and rifampicin (10 mg/ml). Plated oospores were incubated in the dark at 25°C for at least four days before being checked for germination. Germinating oospores were observed using a stereo microscope (60× magnification) and transferred to separate 9 cm 2.5% (v/v) V8 agar plates supplemented with rifampicin (10 mg/ml). Hybrid F_1_ progeny were identified using co-dominant DNA markers that are polymorphic between the parents P6497 and P7064. For production of F_2_ progeny, oospores were generated from F_1_ cultures and isolated as described above. A total of 100 F_2_ progeny were isolated for virulence screening and genetic mapping.

### Plasmid construction, transformation, and plant transient expression assays

For transformation experiments on *P. sojae*, each of the *Avr3a* alleles was amplified from genomic DNA and the resulting PCR products were separated by agarose gel electrophoresis, excised and purified (QIAquick Gel Extraction Kit, Qiagen) prior to cloning. Purified PCR products of *Avr3a* were then subcloned into the stable transformation vector CE+p35, which is a construct of pUC19 with the *Ham34* promoter and terminator of *B. lactucae* [35]. Sub-cloning of *Avr3a* for transcription of the positive (sense) strand was used for overexpression whereas orientation of *Avr3a* for transcription of the negative (anti-sense) strand was used for gene silencing. The CE+p35:Avr3a and a helper plasmid CE+p35:nptII gene (confering resistance to Geneticin) were delivered into *P. sojae* protoplasts by using a polyethylene glycol mediated co-transformation protocol [31], [32]. The transformed cultures were selected for on pea broth agar plates containing 0.8 M mannitol and 30 g/ml Geneticin and propagated in pea broth medium suspended with 30 g/ml Geneticin, and subject to to quantitative real time PCR (qRT-PCR) analysis.

For transient expression by particle bombardment, each of the *Avr3a* alleles were sub-cloned into pFF19 plant transient expression vector containing the 35S promoter. Recombinant plasmids were purified for verification by DNA sequencing and for plant transient expression assays. Particle bombardment assays were carried out using a double-barrelled extension of the Bio-Rad He/1000 Particle Delivery System [31]. To measure the activity of *Avr5* candidates in triggering *Rps*5-dependent cell death, DNA carrying the constructs (1.7 µg per shot) was co-bombarded into soybean leaves along with DNA carrying a GUS reporter gene (1.7 µg per shot). Specific cell death activity was measured as the reduction in the number of blue-staining GUS-positive spots in leaves carrying *Rps*5 compared with leaves lacking either resistance gene. The double-barrelled device was used to deliver a parallel control shot for the expression of GUS DNA plus empty vector (EV) DNA. For each pair of shots, the logarithm of the ratio of the number of blue spots with various *Avr5* constructs to the number with the EV control was calculated. Each assay consisted of eight pairs of shots and was conducted at least twice. The log ratios from all the *Rps*5 leaves were then compared with those from the non-*Rps*5 leaves using the Wilcoxon rank sum test [31].

### Nucleic acid isolation and assay of *Avr5* mRNA levels

Genomic DNA of *P. sojae* was isolated from mycelia grown in 10% vegetable juice (V8) liquid medium according to published methods [6]. Total RNA was isolated using the NucleoSpin RNA II RNA extraction kit (Macherey-Nagel) following the procedures described by the manufacturer. The integrity of total RNA was confirmed using agarose gel electrophoresis.

To investigate the expression efficiency of the *Avr3a^P6497^* transgene in the transformants, qRT-PCR was performed according to the following steps. First-strand cDNA was synthesized using M-MLV reverse transcriptase (RNase-free) and oligo (dT) 18-mer primer (Invitrogen, USA). The qRT-PCR was conducted in 20 µl reactions including 20 ng of cDNA, 0.2 µM gene- specific primer for *Avr5* or the reference *Actin* gene, 10 µl of SYBR *Premix* ExTaq (TaKaRa, Japan), and 6.8 µl of distilled H_2_O. The reactions were performed on a real-time PCR instrument (Applied Bio-systems, PRISM 7300, Foster City, CA, USA) under the following conditions: 95°C for 30 s; 40 cycles of 95°C for 5 s and 60°C for 31 s to calculate cycle threshold values; followed by a dissociation program of 95°C for 15 s, 60°C for 1 min, and 95°C for 15 s to obtain melt curves. Computer software (7300 System Sequence Detection, version 1.4) was used to obtain the expression level of each sample relative to the *Actin* control. Normalization and comparison of mean Ct values were performed as described [36]. The experiment was conducted twice, each with three independent biological replicates.

### Data Deposition

The sequence data for the *Avr3a/5* alleles have been deposited to NCBI GenBank under the accession numbers EF587759, JF412456 and JF433921.

## Supporting Information

Table S1 **List of *P.sojae* strains used in this study.**
(DOC)

Table S2 **Oligonucleotide primers used in this study.**
(DOC)

Figure S1 **Comparison of the** ***Avr3a*** **structural gene in** ***P. sojae*** **strains P6497, P7064, and ACR12, illustrating promoter region insertions and deletions.**
(EPS)

Figure S2 **Representative photographs from co-bombardment and virulence assays performed on soybean plants.** (A) Photographs of soybean L85–3059 (*Rps*5) leaves, after co-bombardment of GUS reporter together with control plasmid or selected *Avr3a* alleles. Numbers in parenthesis indicate total GUS positive spots counted for each treatment, in this experiment (B) Photographs of light-grown soybean plants after inoculation with *P. sojae* wild-type and transformed strains, as indicated. (C) Photographs of etiolated soybean hypocotyls after inoculation with *P. sojae* wild-type and transformed strain, as indicated.
(TIF)
