# Supplementary material for: Sequence Variants of the Phytophthora sojae RXLR Effector Avr3a/5 Are Differentially Recognized by Rps3a and Rps5 in Soybean
Source: PLoS One. 2011 Jul 14;6(7):e20172. doi: 10.1371/journal.pone.0020172 (PMC3136461; doi:10.1371/journal.pone.0020172)
Supplement: Table S1 — List of P.sojae strains used in this study. (DOC) [file pone.0020172.s001.doc]

| **Table S1. List of *Phytophthora sojae* strains used in this study** | | | | | |
| --- | --- | --- | --- | --- | --- |
| Race | Name | Virulence^a^ | | Origin^b^ | From^c^ |
|  |  | *Rps*5 | *Rps*3a |  |  |
| race 1 | 48FPA18 | A | A | Ohio (F.S.) | F.S. |
| race 2 | P6497 | A | A | Mississippi (B.K.) | B.M.T. |
| race 3 | 25MEX4 | A | A | Ohio (F.S.) | F.S. |
| race 7 | P7064 | V | V | Canada (C.M.) | B.M.T. |
| race 8 | ACR8 | A | A | Unknown | T.A. |
| race 9 | ACR9 | A | A | Harrow, ON (C.M.) | T.A. |
| race 10 | ACR10 | V | V | Stoneville, MS (B.K.) | T.A. |
| race 11 | ACR11 | A | A | Harrow, ON (C.M.) | T.A. |
| race 12 | ACR12 | V | A | Stoneville, MS (B.K.) | T.A. |
| race 16 | ACR16 | V | V | Stoneville, MS (B.K.) | T.A. |
| race 17 | P7074 | V | V | Stoneville, MS (B.K.) | T.A. |
| race 19 | P7076 | V | A | Stoneville, MS (B.K.) | B.M.T. |
| race 20 | ACR20 | V | A | Unknown | T.A. |
| race 21 | ACR21 | V | V | Lafayette, IN (F.A.L) | T.A. |
| race 24 | ACR24 | A | V | Lafayette, IN (F.A.L) | T.A. |
| race 25 | ACR25 | A | A | Lafayette, IN (F.A.L) | T.A. |

^a^Virulence on soybean Rps genes; A, avirulent; V, virulent.

^b^Original site of isolation and where known, investigator who conducted the isolation.

^c^Investigator who provided the culture to Agriculture and Agri-Food Canada, London, ON. T.A. = T. Anderson (Agriculture and Agri-Food Canada, Harrow, ON); B.K.= B. Keeling (USDA, Stoneville, MS); F.A.L.= F.A. Laviolette (Purdue University, West Lafayette, IN); C.M = C. Meharg (Agriculture and Agri-Food Canada, Harrow, ON); F.S.= F. Schmitthenner (Ohio State University, Wooster, OH); B.M.T.= B. M. Tyler, (Virginia Bioinformatics Institute, Blacksburg, VA); E.W. = Ed Ward (Agriculture and Agri-Food Canada, London, ON).
