# Supplementary material for: Sequence Variants of the Phytophthora sojae RXLR Effector Avr3a/5 Are Differentially Recognized by Rps3a and Rps5 in Soybean
Source: PLoS One. 2011 Jul 14;6(7):e20172. doi: 10.1371/journal.pone.0020172 (PMC3136461; doi:10.1371/journal.pone.0020172)
Supplement: Table S2 — Oligonucleotide primers used in this study. (DOC) [file pone.0020172.s002.doc]

| **Table S2.** Oligonucleotide primers used in this study. | | | |
| --- | --- | --- | --- |
| Target gene or marker name | Primer name | Sequence, 5’ to 3’ | Application |
| *Avh*36 | Avh36S-F  Avh36S-R | TGGTTAACAAAGAGCGAAGTAG  CTACCTCCAGTTGACATGTTC | To sequence *Avh*36 polymorphism |
| *Avh*36 | Avh36S-R  Avh36-F  Avh36-R | CTACCTCCAGTTGACATGTTC  GACTCTCCCTCGATTTTGTG  CTAAGTGAGGCCCCTCTCC | RT-PCR transcript detection |
| *Avh*38 | Avh38-SF  Avh38-SR | GGGCTGATGCGGAGACTC  CGTACACGTTCGCCATCTTG | To sequence *Avh*38 polymorphism |
| *Avh*38 | Avh38-F  Avh38-R | ATGCGTGCCCAGTACTTCCT  CGTGTCGGATGTGCCTTAG | RT-PCR transcript detection |
| *Avr3a^P6497^* | CE+p35-F  CE+p35-R | TTGAAAACAATCGTTCTTTCAC  CTGTTTGTGGGGTTGTGAAG | PCR of genomic DNA |
| *Avr3a^P6497^* | Avr3a-qRT-F  Avr3a-qRT-R | TCGCTCAAGTTGTGGTCGTC  TCGACAGCGTCCTATCTTCG | Quantitative real time PCR |
| *ActinA* | actAF1  actAF2 | ACTGCACCTTCCAGACCATC  CCACCACCTTGATCTTCATG | Quantitative real time PCR |
| 8R  scaffold_80: 236500-239000 | R8F3  R8new | CAATTACGAGCTCCAACATGT  TCCATTACAACGCATCTCCTG | *Xmn*I digestion of PCR product for genetic mapping  P6497: 693 bp  P7064: 179 + 514 bp |
| NS2  scaffold_80: 331500-333200 | R2-2F  R2-2R | GTGCTGAGCGTGGAGTTACG  GCCGAGATGAATCCCTTGAG | *Dpn*II digestion of PCR product for genetic mapping  P6497: 843 bp  P7064: 235 + 608 bp |
