# Supplementary figures and images for: Sequence Variants of the Phytophthora sojae RXLR Effector Avr3a/5 Are Differentially Recognized by Rps3a and Rps5 in Soybean
Source: PLoS One. 2011 Jul 14;6(7):e20172. doi: 10.1371/journal.pone.0020172 (PMC3136461; doi:10.1371/journal.pone.0020172)

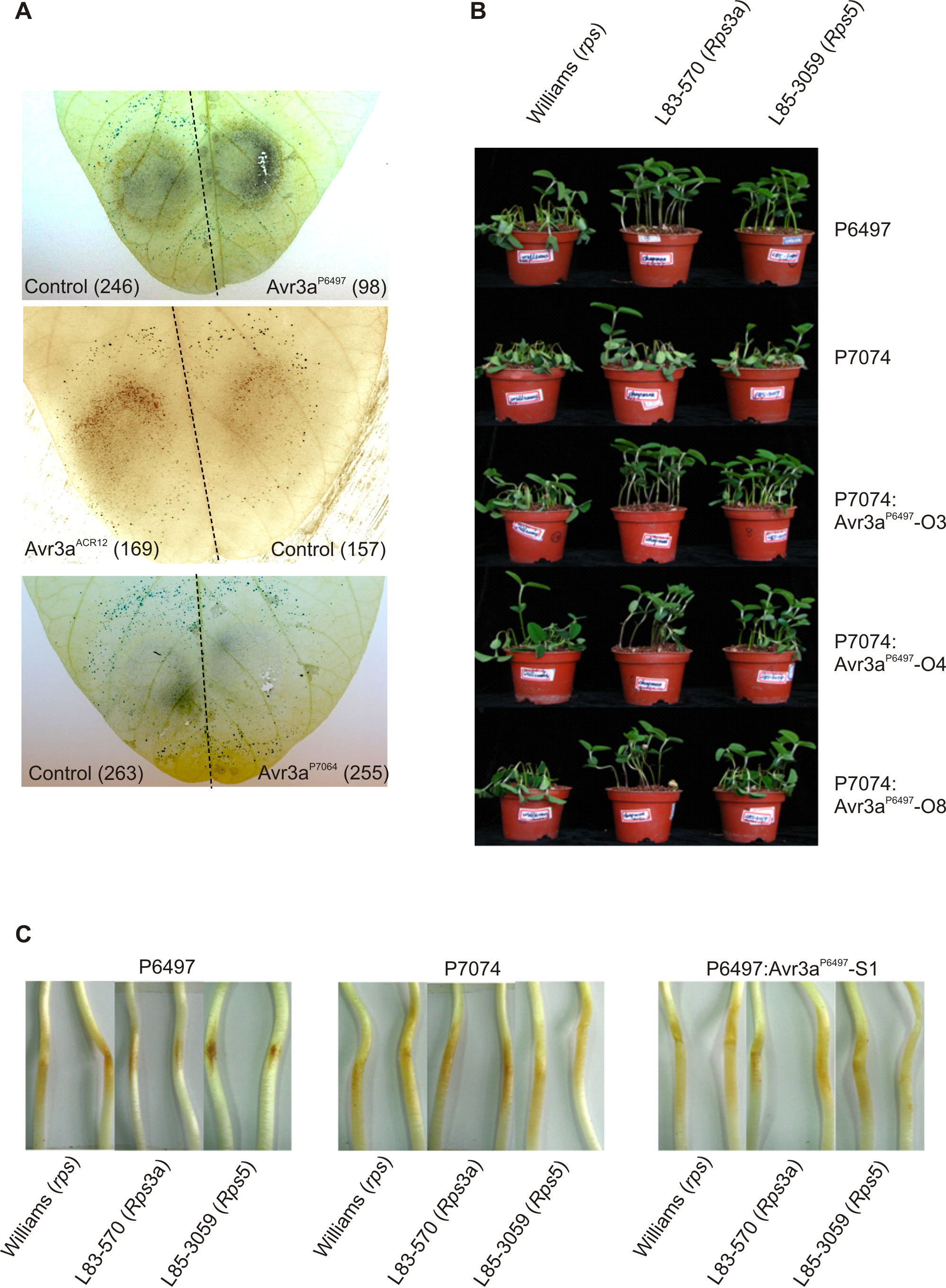

Supplement: Figure S2 — Representative photographs from co-bombardment and virulence assays performed on soybean plants. (A) Photographs of soybean L85–3059 (Rps5) leaves, after co-bombardment of GUS reporter together with control plasmid or selected Avr3a alleles. Numbers in parenthesis indicate total GUS positive spots counted for each treatment, in this experiment (B) Photographs of light-grown soybean plants after inoculation with P. sojae wild-type and transformed strains, as indicated. (C) Photographs of etiolated soybean hypocotyls after inoculation with P. sojae wild-type and transformed strain, as indicated. (TIF) [file pone.0020172.s004.tif]
